# Effects of Variety, Plant Location, and Season on the Phyllosphere Bacterial Community Structure of Alfalfa (*Medicago sativa* L.)

**DOI:** 10.3390/microorganisms10102023

**Published:** 2022-10-13

**Authors:** Mingzhu Zhang, Chao Peng, Wentao Sun, Rui Dong, Jun Hao

**Affiliations:** College of Animal Science, Guizhou University, Guiyang 550025, China

**Keywords:** phyllosphere, bacteria diversity, environmental factor, microbiome, alfalfa

## Abstract

Plant phyllosphere bacteria are vital for plant health and productivity and are affected by both abiotic and biotic factors. In this study, we surveyed the structure of the phyllosphere bacterial community associated with alfalfa. For two varieties of alfalfa, forty-eight samples of phyllosphere communities were collected at two locations over four seasons in 2020. Proteobacteria and actinobacteria were associated with the dominating phylum in the bacterial communities of the alfalfa phyllosphere. *Sphingomonas* was the most abundant genus-level bacteria, followed by *Methylobacterium*, *Burkholderia-Caballeronia-Paraburkholderia*, and *Pseudomonas*. Sampling time had a greater affect than site and variety on alfalfa surface microorganisms. The variation in phyllosphere bacterial community assembly was mostly explained by the season–site interaction (43%), season–variety interaction (35%), and season (28%). Variety, site–variety interaction, and season–site–variety interactions did not have a meaningful effect on phyllosphere bacterial diversity and community structure. The bacterial community in the phyllosphere of alfalfa showed seasonal changes over time. The environmental factors that contributed most to the phyllosphere bacterial community of alfalfa were temperature and sunshine duration, which were significantly positively correlated with most of the dominant bacterial genera in the alfalfa phyllosphere.

## 1. Background

The entire outer surface of the aboveground portion of a plant is referred to as the phyllosphere. This surface offers a habitat favorable for the survival of some microorganisms [1]. There are rich and diverse microbial communities in the phyllospheres of forage plants, mainly dominated by bacterial communities [2]. On average, the phyllosphere can support 10^6^~10^7^ bacterial cells per square centimeter, with bacteria occupying a major position in the phyllosphere microbial community [3]. These phyllosphere microorganisms are involved in the physiological metabolism of forage grasses through interactions with the forage host [4]. They play an important role in the global carbon and nitrogen cycles and have not only a complex relationship with plant hosts but also various functions, such as fixing nitrogen [5], promoting forage growth [6], enhancing forage resistance [7], changing forage surface properties, and degrading pesticide residues on forage surfaces [8,9].

The phyllosphere environment is a very demanding and unstable habitat for phyllosphere microorganisms, which live on the plant surface and are often exposed to water and nutrient deficiencies as well as drastic environmental changes, including bright light, high temperatures [10], and high UV exposure [11]. In addition, biological factors such as plant species and plant physiological status also affect phyllosphere microorganisms [12]. Different plants can selectively accumulate various microorganisms in the rhizosphere, leaf space, and inner layers. Due to the significant role that plant-associated microbiota play in the regulation of numerous biological processes, as well as their influence on a variety of traits involved in plant growth and development and plant responses to unfavorable environmental conditions, genetic variation within and between species can also affect the microbiome composition of crops [13,14]. Over the years, molecular surveys have shown that plant phyllospheres contain diverse bacterial communities [15,16,17]. These communities are also known to vary in their spatial [18,19] and temporal patterns from molecular and enrichment approaches [20]. The microorganisms may have certain distribution characteristics among different regions. This, in turn, is related to climate, which can influence the growth of plant phyllosphere microorganisms by determining the environmental conditions (soil, water, air, heat, etc.) in a region. For example, *Penicillium*, *Aspergillus*, and *Fusarium* are more likely to be found in the plant phyllosphere under hot and humid growing conditions [21]. The findings of one study revealed that the host species identity (27%) and species–site interaction (14%), location (11%), and season (1%) contribute to the variation in the composition of the phyllosphere bacterial population [22]. However, it has also been shown that the contribution of planting sites to the effect of phyllosphere microorganisms is stronger than that of varieties [23]. Although earlier research has revealed that phyllosphere communities are more complicated than previously thought, there are still many important issues to be resolved regarding their geographical and temporal variability [24,25]. Phyllosphere bacteria assembly drivers have been measured in earlier research [26,27], but the majority of these studies looked at only one possible driver of phyllosphere community structure. Additionally, prior research has concentrated on the study of microorganisms in a fixed part of the plant, such as the foliage [23,28] or rhizosphere [29]; however, studies on the effect of epiphytic bacteria on the plant as a whole are lacking.

Alfalfa is well-known as a high-quality forage worldwide and is often used for fresh feed, hay, and silage. Silage is the end result of an anaerobic fermentation process powered by bacteria discovered on the crop at harvest. The main phyllosphere microorganisms of forage are lactic acid bacteria, *Clostridium*, *Acetobacter*, *Bacillus*, and other aerobic bacteria [30]. The most advantageous microorganisms for boosting silage fermentation are lactic acid bacteria because they produce lactic acid and provide an acidic environment that prevents the growth of other dangerous microorganisms [31]. *Enterobacteriaceae*, *Acetobacter*, *Bacillus*, *Clostridium*, and *Listeria* are considered unfavorable bacteria for silage fermentation [32] For example, *Acetobacter* oxidizes lactic and acetic acid, leading to an increase in pH and a loss of dry matter in the form of carbon dioxide [31]. Changes in microorganisms during fermentation depend on the characteristics of the crop and the silage technology used, and these changes can diversify the fermentation process and thus affect the quality of silage fermentation. However, the effects of various environmental factors on the phyllosphere microorganisms of alfalfa have not been considered from a silage perspective. Therefore, we predicted that the bacterial community in the alfalfa phyllosphere would be significantly influenced by variety, plant location, and season. The purpose of this study was to investigate the ecological factors that contribute to variation in the bacterial community composition of the alfalfa phyllosphere. Our goals were to: (1) identify the phyllosphere bacteria found in alfalfa and (2) quantify the relative importance of three factors on the composition of phyllosphere bacterial communities: variety, site, and season.

## 2. Materials and Methods

### 2.1. Study Site

**Figure 1:**
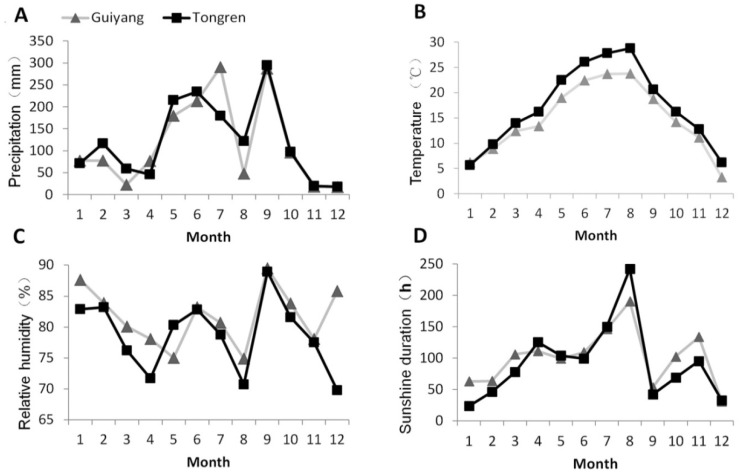
The monthly average changes in precipitation (**A**), temperature (**B**), relative humidity (**C**), and sunshine duration (**D**) in the areas of Tongren and Guiyang in 2020.

The study plots were located in two cities in Guizhou Province, China: Guiyang, at the Guizhou University pilot site (106°07′ E; 26°11′ N; elevation 1100 m), and Tongren (108°20′14″ E; 27°32′03″ N; elevation 614 m). The distance between sites is 248 km. Both experimental sites had not been planted with alfalfa, the Guiyang experimental site had not been planted with crops in the early stage, mainly wild weeds, and the Tongren experimental site had corn as the previous crop. The data of soil basic nutrients in Guiyang and Tongren experimental sites were provided by the research group of Mr. Dong of Guizhou University (Appendix A) [33]. Two alfalfa varieties, *Medicago sativa* L. cv. Xinjiang Daye, and *Medicago sativa* L. cv. Algonguin, which are suitable for growing in acidic soils in the humid and hot regions of southwest China, were selected for planting in each trial field. Each site had 3 plots, and 60 individual plants of each variety were planted in each plot, with 50 cm intervals between plant rows. Alfalfa was planted at the start of 2018, mown six times a year from 2019 onward, and sampled at the initial flowering stage prior to mowing. No fertilization and artificial irrigation treatments were conducted. The experimental field of Guizhou University in Guiyang has a subtropical humid monsoon climate, with obvious plateau climate characteristics. The experimental field in Tongren belongs to the central subtropical humid monsoon climate, with sufficient sunshine and abundant rainfall. We obtained monthly climate data from the China National Meteorological Information Center (Figure 1).

### 2.2. Bacterial Community Collection

A total of 48 samples were collected. We sampled phyllosphere bacteria from these two varieties of alfalfa (Xinjiang Daye and Algonquin) at two sites (Guiyang and Tongren) during four seasons (January, April, July, and October) in 2020. Each site contained three planting plots, five sampling points were randomly selected within each plot (each 2 m apart), and three alfalfa plants were randomly selected for sampling at each sampling point. Each alfalfa plant was swabbed with 5 swabs, and 75 swabs per plot were collected together as one sample for bacterial sequencing. The cotton swab sampling method was used for bacterial community collection [34]. Sterile cotton swabs (Wela Biotechnology Co., Ltd., Guiyang, China) were moistened with sterilized saline (0.9% concentration) and wiped on the outer surface of the aboveground portion of the alfalfa (stems, leaves) for 10 s. The heads of the swabs were collected and quickly placed in sterilized centrifuge tubes, snap-frozen in liquid nitrogen, and brought back to the laboratory for storage at −80 °C. 

### 2.3. Microbiome Analysis

Using a HiPure Soil DNA Extraction Kit, microbial DNA was isolated (Magen, Guangzhou, China). Using the following parameters, the 16S rRNA gene V3–V4 region was amplified with PCR: 94 °C for 2 min, 98 °C for 10 s, 62 °C for 30 s, and 68 °C for 30 s for 30 cycles, and lastly 68 °C for 5 min. This process was accomplished using the primers 341F: CCTACGGGNGGCWGCAG and 806 R: GGACTACHVGGGTATCTAAT [35]. PCRs were carried out three times. The following was the composition of the amplification system: 50 L of a mixture of 5 L of KOD buffer (10 M), 5 L of 2 mm dNTPs, 3 L of 25 mm MgSO4, 1.5 L of forward and reverse primers (10 M), 1 L of KOD polymerase (TOYOBO, Osaka, Japan), and 100 ng of template DNA.

Using the AxyPrep DNA Gel Extraction Kit (Axygen Biosciences, Union City, CA, USA), amplicons were extracted from 2% agarose gels, purified in accordance with the manufacturer’s instructions, and quantified using the ABI StepOnePlus Real-Time PCR System (Life Technologies, Foster City, CA, USA). On an Illumina platform, the purified amplicons were sequenced in accordance with best practices, then paired-end sequencing (PE250) was carried out in accordance with best practices.

The clean readings, which had a minimum overlap of 10 base pairs and a maximum mismatch rate of 2%, were combined into tags using FLASH [36] (version 1.2.11) via the following procedure: when the number of bases in the continuous low-quality value (the default quality threshold) reached the predetermined length (the default length is 3 bp), raw tags from the first low-quality base site were broken, and tags with a continuous high-quality base length less than 75% of the tag length were filtered. Using the UCHIME technique, tags’ chimeras (for 16S sequencing analysis) were examined [37]. For further study, the clean tags that were acquired after filtering the chimeras were employed. Using UPARSE [38] (version 9.2.64), the clean tags were organized into operational taxonomic units (OTUs) based on a 97% similarity threshold. As the representative sequences for each OTU, the tag sequences with the highest abundance were chosen. Representative OTU sequences and the SILVA [39] database were compared (version 132).

### 2.4. Statistical Analysis

Species abundance chorograms, i.e., presented in a circular layout, were plotted using circus software [40] (version 0.69-3). The vegan package in R (version 2.5.3) was used to perform beta diversity analysis for NMDS multivariate statistical analysis based on Bray–Curtis distances. For Bray–Curtis distances, the PERMANOVA test was used. R (version 2.5.3)’s vegan package was used to perform redundancy analysis (RDA) to elucidate how environmental factors will affect community composition in 2020. Using the R language’s psych package, the Pearson’s correlation coefficients between environmental parameters and species were determined (version 1.8.4). Utilizing the free online platform of Omicshare tools, the data were evaluated.

## 3. Results

### 3.1. Phyllosphere Bacterial Diversity and Bacterial Community Dynamics

**Figure 2:**
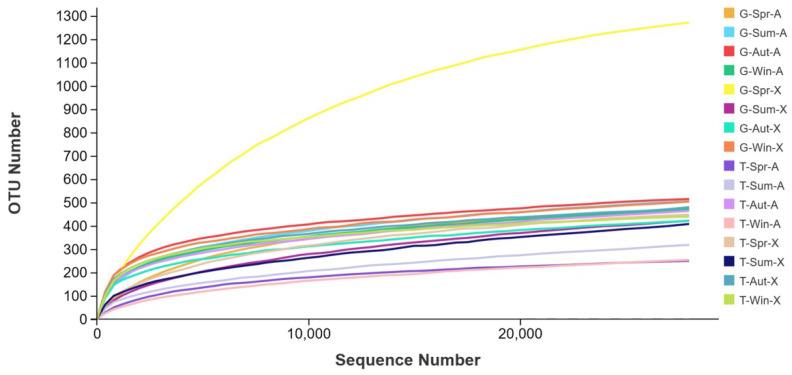
Rarefaction curves of OTUs across different samples.

A total of 4,997,132 high-quality sequences were produced after quality filtering and the elimination of nonbacterial sequence reads. As seen in Figure 2, as the quantity of sequencing data reached approximately 20,000, the rarefaction curve of each sample tended to approach the plateau phase. It was clear from this that there were enough sequences in these samples to adequately describe the bacterial composition.

In the alfalfa samples, sequencing revealed 7393 bacterial operational taxonomic units (OTUs, binned at 97% similarity). The sampling depth effectively captured the majority of the bacterial communities, as shown by the Good’s coverage of all samples, which was roughly 0.99. The OTUs, Shannon index, Chao index, and Ace index tended to increase from spring to autumn and then decrease from autumn to winter in all alfalfa samples, except for the Xinjiang Daye group grown in Guiyang, but these trends were not significant (Table 1).

**Figure 3:**
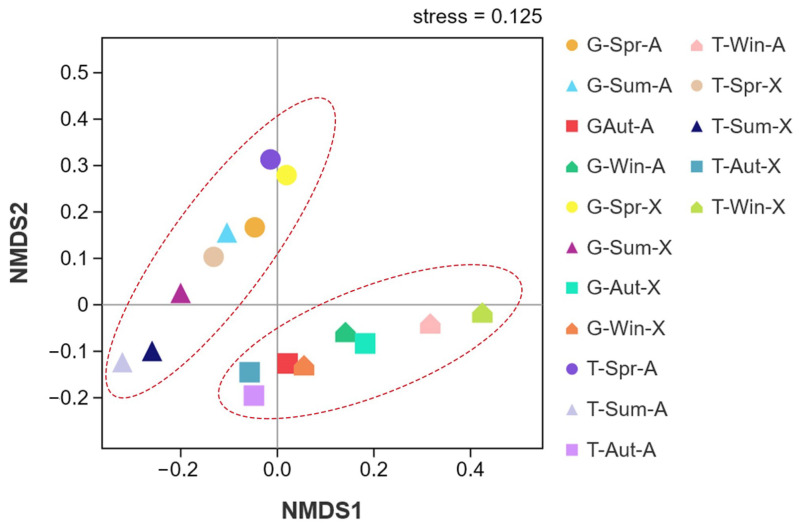
Non-metric multi-dimensional scaling (NMDS) plot of bacterial community structures based on Bray–Curtis differences. G, Guiyang; T, Tongren; Spr, spring; Sum, summer; Aut, autumn; Win, winter; A, Algonguin; X, Xinjiang Daye.

The bacterial community structures of all alfalfa samples were illustrated using an NMDS plot based on the Bray–Curtis distance (Figure 3). The samples in the seasons were obviously distinguished, indicating that the alfalfa phyllosphere had similar bacterial communities at each season regardless of the variety and plant location. However, Figure 3 shows that the different varieties of alfalfa in Guiyang and Tongren exhibited overlap to a certain extent in spring and summer and a certain extent in autumn and winter.

**Figure 4:**
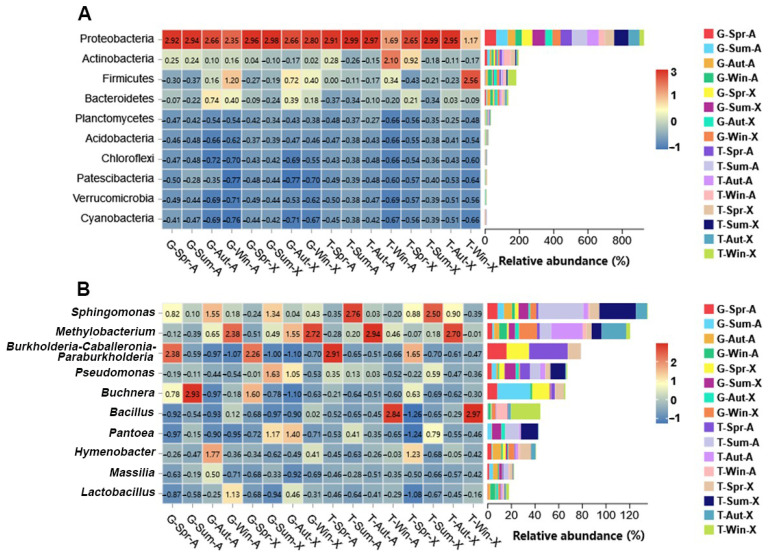
Phyllosphere bacterial communities at the phylum level (**A**) and genus level (**B**). G, Guiyang; T, Tongren; Spr, spring; Sum, summer; Aut, autumn; Win, winter; A, Algonguin; X, Xinjiang Daye. The legend shows the abundance values after normalization for the relevant phylum and genus.

The relative abundance of the alfalfa phyllosphere bacterial microbiome is shown in Figure 4. The 16S ribosomal DNA analysis demonstrated that, according to the composition of the phyllosphere community, Proteobacteria and Actinobacteria were the most dominant bacterial phyla. From spring to winter, Proteobacteria showed an increasing and then decreasing trend in all taxa and reached maximum relative abundance during the summer. The relative abundance of Actinobacteria showed a decreasing and then increasing trend for Algonguin in the four seasons at the two sites; however, a continuous decreasing trend was observed in Xinjiang Daye. In addition, many other phyla, such as Bacteroidetes (1.10% to 19.22%), Firmicutes (2.50% to 47.29%), and Planctomycetes (0.31% to 5.35%), were identified. Their relative abundance in all samples showed variable changes.

The relative abundance of the top 10 bacterial genera in the samples is shown in Figure 4B. *Sphingomonas*, *Methylobacterium, Burkholderia-Caballeronia-Paraburkholderia*, and *Pseudomonas* were the top genera. Among them, *Sphingomonas*, as a dominant genus, appeared more abundant in summer, and *Methylobacterium* showed higher relative abundance in winter in Guiyang and in autumn in Tongren.

### 3.2. Drivers of Variation in Phyllosphere Bacterial Community Composition and Diversity

According to a PERMANOVA of community structure variation, the interaction between season and site explained 42.9% of the variation in bacterial communities, the interaction between season and variety explained 34.7%, season explained 27.6%, and site explained 4.0%. However, neither variety, nor the interaction of variety and site, nor the interaction of season, site, and variety had a significant influence on the diversity and community structure of phyllosphere bacteria (Table 2).

**Figure 5:**
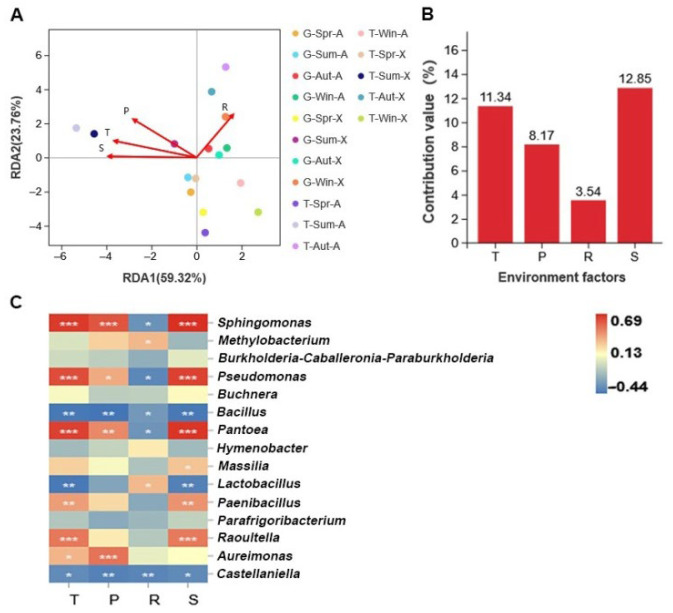
Correlations between environmental factors and community composition. (**A**) Redundancy analysis (RDA) of microbial community composition and environmental factors. S: sunshine duration, P: precipitation, R: relative humidity, and T: temperature. (**B**) Variance partitioning analysis (VPA) of the contribution of environmental factors to the total variation in genus distribution value (%). (**C**) Heat map of environmental factors and microbes based on the Pearson correlation. Significance levels are indicated by *, *p* < 0.05; **, *p* < 0.01; ***, *p* < 0.001.

Examining the relationship between microorganisms and environmental factors is useful for comprehending the mechanism of phyllosphere community assembly of alfalfa. Distance-based redundancy analysis (db-RDA) and a correlation analysis between microbiota and driving factors were carried out to clarify the primary environmental drivers in various alfalfa phyllospheres under various groups, as shown in Figure 5A. The results showed that the explanatory rate of environmental factors for the distribution of bacterial communities was 83.08%. This indicated that environmental factors had an important impact on the phyllosphere microorganisms of alfalfa. In addition, temperature, sunshine duration, precipitation, and relative humidity had a strong correlation with the microbial community (*p* < 0.05). Precipitation, temperature, and sunshine duration were positively correlated with most alfalfa bacterial communities sampled in summer. The relative humidity was strongly correlated with the phyllosphere bacterial community of alfalfa sampled in autumn and winter.

We used Pearson coefficients to investigate the correlations between specific genera and driving factors to better understand these linkages (*p* < 0.05; Figure 5C). Figure 5C shows that the genera *Sphingomonas*, *Pseudomonas*, and *Pantoea* were positively correlated with temperature, precipitation, and sunshine duration. This explains the higher relative abundance of these three bacterial genera in summer than in other seasons. We also found that temperature, precipitation, and sunshine duration were negatively correlated with *Bacillus*, and relative humidity was negatively correlated with *Sphingomonas*, *Pseudomonas*, *Pantoea*, and *Bacillus*. The environmental factors that contributed most to the phyllosphere bacterial community of alfalfa were temperature and sunshine duration (Figure 5B).

## 4. Discussion

### 4.1. Analysis of Bacterial Community Composition and Diversity

According to DNA sequencing, Proteobacteria were the most prevalent phylum, followed by Actinobacteria. The most prevalent phyllosphere bacteria in a variety of crops, including miscanthus [41], thale cress [42], tea [43], and rice [44], are Proteobacteria and Actinobacteria. Proteobacteria are able to colonize a variety of habitats, including the rhizosphere and phyllosphere, which could account for their predominate presence [45].

The relative abundance of dominant genera of alfalfa phyllosphere bacteria in different locations, varieties, and sampling times is not very high. This might be because the phyllosphere microbial community is also characterized by a low number of highly abundant microbial taxa and a high number of rare taxa with low relative abundance [3,46]. The major bacteria at the genus level were *Sphingomonas*, *Methylobacterium*, *Burkholderia-Caballeronia-Paraburkholderia*, and *Pseudomonas*. All these genera were found to be dominant in different plant phyllospheres [19,47,48]. Notably, differences still existed, while certain dominant bacterial genera appear to be the same in other studies on alfalfa phyllosphere bacteria. Mc Garvey et al. [49] found that the main alfalfa phyllosphere genera were *Erwinia*, *Escherichia*, *Pseudomonas*, *Pantoea*, and *Enterobacter*, while Zheng et al. [50] found the main bacterial genera to be *Pantoea*, *Enterobacter*, and *Buchnera*. In combination with our experimental results, the same suggestion was made that different seasons, sites, and varieties can have an effect on the composition of the alfalfa phyllosphere bacterial community. From a production point of view, *Pseudomonas* [51], *Methylobacterium* [52,53], and *Bacillus* [54] were recognized as having the potential to promote plant growth. It has been found that *Bacillus* and *Pantoea* suppress fungal growth and are tolerant to conditions such as low water potential and osmotic stress in a study on the biocontrol potential of phyllospheric microorganisms against *Exserohilum turcicum* [55]. 

On the leaves of *Arabidopsis thaliana*, *Sphingomonas* inhibit the growth of pathogenic *Pseudomonas syringae* pv. *syringae* DC3000 and suppress disease symptoms [56]. These findings suggested that the dominant phyllosphere microorganisms are beneficial to the health and productivity of alfalfa. In the production of silage, *Pseudomonas* is an unfavorable bacterium that can thrive in an anaerobic environment and therefore is harmful for silage production. The biogenic amine produced by *Pseudomonas* leads to a decrease in protein content, which leads to a decrease in the nutritional quality of the feed [57]. Additionally, *Pseudomonas* is the primary group of foodborne and hazardous bacteria linked to fresh produce [58]. In our study, *Pseudomonas* showed higher relative abundance in summer and was positively correlated with temperature and sunshine duration. Therefore, we speculated that alfalfa harvested in the hot and rainy summer was not conducive to silage fermentation. Notably, the relative abundance of bacteria favoring silage fermentation, such as *Lactococcus*, *Lactobacillus*, and *Weissella*, accounted for less than 1% in all our samples. Lactobacillales (*Lactobacillus*, *Lactococcus*, and *Weissella*) are the main heterofermenters in the silage process, inhibiting negative microorganisms and promoting fermentation through the production of metabolites such as organic acids, bacteriocins, and competition for nutrients [59]. Our results show that the alfalfa phyllosphere lacks beneficial bacteria favoring fermentation. Therefore, microbial additives should be used to promote the fermentation of alfalfa before silage.

The phyllospheres of the two sites and varieties of alfalfa showed some degree of overlap in spring and summer and some degree of overlap in autumn and winter (Figure 3). This indicates that the distribution and structure of bacterial communities in alfalfa differed less in these two periods. The overlap may have been due to the small changes in environmental factors such as temperature, sunshine duration, precipitation, and relative humidity.

### 4.2. Influence of Variety, Site, and Season on the Structure of Alfalfa Phyllosphere Bacterial Communities

Abiotic factors such as environmental conditions (solar UV radiation, temperature fluctuations, relative humidity, and nutrient availability), agronomic practices, and biotic factors such as host plant species all influence the structure of the phyllosphere bacterial community [3,60,61,62]. Using 16S rRNA gene sequencing, this study investigated the effects of alfalfa variety, plant location, and season on the composition and diversity of alfalfa phyllosphere bacterial communities. When compared to site and variety, season was the most important driver of the phyllosphere bacterial community structure in alfalfa (R^2^ = 27.6%). This result is similar to previous findings that show that annual changes have the strongest effect on bacterial communities [63]. The composition of the tree phyllosphere can vary considerably over longer time periods, with temporal variation on a given tree exceeding the variation in community composition among individual trees sampled on a given day, and can exhibit certain seasonal patterns [64]. Although there are other studies reporting different plant materials with different phyllosphere microbiota [22,65], little variation in bacterial community composition has been observed in different species of plants compared to other plant types [18,23]. According to Kim et al. [66], the phylogeny of the host plant determines how similar the phyllosphere bacterial communities of these tree species are to one another, and more similar communities are found in closely related host plants. The selection impact of host plants reduces bacterial diversity, according to Xiong et al. [45]. The bacterial populations in the tobacco phyllosphere have also shown significant resistance to environmental perturbations, according to Chen et al. [67]. These results might suggest that the same host plant exhibits similar bacterial community structures, in which case the impact of different alfalfa types on the bacterial community structure would not be very noteworthy. We also found that the interaction of season and site had the greatest influence on the community structure of alfalfa phyllosphere bacteria (R^2^ = 42.9%). However, neither variety, nor the interactions of site and variety, nor the interactions of season, site, and variety significantly impacted the richness and community structure of phyllosphere bacteria. Numerous studies have documented the clear impact of distance on bacterial diversity, which is primarily attributable to site-specific elements such as nutrient availability, temperature swings, relative humidity, and sun radiation [45]. Additionally, Redford et al. [68] hypothesized that each type of tree has a unique structural composition of phyllosphere bacterial population that remains constant even after the trees are planted in various locations throughout the globe. This may help to explain why, in our investigation, the interaction between cultivation sites and variety had no appreciable impact on the diversity and community structure of the phyllosphere.

### 4.3. Correlation Analysis between Bacterial Communities and Environmental Factors

Season and location interactions significantly affect alfalfa surface microorganisms, and this effect may be caused by changes in sunshine duration, temperature, and precipitation [69]. In this study, *Sphingomonas*, *Pseudomonas*, and *Pantoea* were positively correlated with temperature, precipitation, and sunshine duration (*p* < 0.01), and the dominant bacterial genera on alfalfa samples were *Sphingomonas* and *Pseudomonas*. This indicated that the main factors affecting the microbial diversity of the alfalfa phyllosphere were temperature, precipitation, and sunshine duration. In addition, the three aforementioned genera were positively correlated with sunshine duration but negatively correlated with relative humidity. This indicates that they are suitable for growth in hot and dry environments. This result is different from those of Guan et al. [70], who suggested that *Sphingomonas* is suitable for growth in cool and humid environments. Proteobacteria comprises diazotrophic bacteria that can use atmospheric dinitrogen as a source of nitrogen [71]. The richness of N_2_-fixing bacteria increased with drought, suggesting that their supplementation may expand plant adaptation to the environment [72]. *Sphingomonas* [73], *Pseudomonas* [74], and *Pantoea* [75] all belong to Proteobacteria and have a nitrogen-fixing role, which may explain why these bacterial genera were negatively correlated with relative humidity in our study. Temperature, precipitation, and sunshine duration were negatively correlated with *Bacillus*, which explains why *Bacillus* was more abundant in winter than in other seasons. *Bacillus* are the main spoilage bacteria in silage, mainly degrading proteins and amino acids, and play an important role in silage spoilage deterioration under aerobic conditions [76]. Our research revealed that the bacterial community of the phyllosphere varied, possibly as a result of growing season disturbances brought on by UV exposure, moisture levels, resource availability, or changes in leaf cuticle characteristics. As a result, a “climax” community was not formed, and neither bacterial diversity nor community composition stabilized over time [77].

## 5. Conclusions

In this study, we described the bacterial communities found in the phyllosphere of alfalfa and investigated the effects of sample site, season, and variety on phyllosphere community structure. Our main conclusions are that season–site interaction, season–variety interaction, and season accounted for the majority of the variation in phyllosphere bacterial community formation. The environmental factors that contributed most to the phyllosphere bacterial community of alfalfa were temperature and sunshine duration, which were significantly positively correlated with most of the dominant bacterial genera in the alfalfa phyllosphere. The bacterial community in the phyllosphere of alfalfa shows a certain seasonal change over time and high sensitivity to changing environmental parameters. In addition, the alfalfa phyllosphere lacks beneficial bacteria that can initiate silage fermentation. Thus, the addition of exogenous substances to alfalfa silage is recommended to promote its fermentation.

## Figures and Tables

**Table 1:** The sequencing index of samples based on 16S rRNA gene analysis.

Site	Variety	Season	OTUs	Shannon	Chao	Ace	Coverage
Guiyang	Algonguin	Spring	592	6.35 bcd	563.03 ab	549.21 b	0.999
		Summer	717	4.99 ab	952.40 ab	992.39 ab	0.997
		Autumn	740	6.77 a	1005.85 ab	1047.94 ab	0.998
		Winter	690	7.10 abc	991.58 ab	1015.89 ab	0.998
	Xinjiang Daye	Spring	1384	6.65 ab	1353.79 a	1399.40 a	0.997
		Summer	692	5.10 bcd	956.60 ab	986.77 ab	0.997
		Autumn	637	6.88 ab	913.76 ab	948.01 ab	0.998
		Winter	734	6.72 a	1077.28 ab	1112.48 ab	0.998
Tongren	Algonguin	Spring	277	5.18 cd	292.41 b	292.21 b	0.998
		Summer	552	4.14 abc	917.46 ab	914.48 ab	0.998
		Autumn	696	6.45 ab	1022.10 ab	1057.75 ab	0.998
		Winter	434	4.84 d	628.20 ab	650.52 ab	0.998
	Xinjiang Daye	Spring	595	5.66 abcd	605.82 ab	615.97 ab	0.999
		Summer	682	4.72 abc	964.86 ab	986.18 ab	0.997
		Autumn	674	6.72 ab	939.24 ab	940.96 ab	0.998
		Winter	585	5.89 ab	782.16 ab	797.57 ab	0.998

OTUs, number of operational taxonomic units. Coverage, Good’s coverage. Means with different letters in the same column (a–d) are significantly different (*p* < 0.05).

**Table 2:** Bacterial community structure variation explained by various factors (PERMANOVA on Bray–Curtis dissimilarities).

Variables		Bray–Curtis Dissimilarities	
		R2 (%)	*p* (>F)
Single factor	Season	27.6	0.001 ***
	Site	4.0	0.004 **
	Variety	NS	NS
Second-order interaction	Season * site	42.9	0.001 ***
	Season * variety	34.7	0.001 ***
	Site * variety	NS	NS
Third-order interaction	Season * site * variety	NS	NS

Significance levels for each variable are given as follows: *, *p* < 0.05; **, *p* < 0.01; ***, *p* < 0.001; NS, *p* > 0.1.

## Data Availability

The datasets used and/or analyzed during the current study are available from the corresponding author upon reasonable request.

## Appendix A

**Table A1:** Soil physical and chemical properties in the two study areas.

Research Area	pH	AN mg/kg	TN mg/kg	TK g/kg	TP g/kg	AK mg/kg	TOC g/kg	AP mg/kg	AMn mg/kg	AFe mg/kg	ACu mg/kg	AZn mg/kg
Guiyang	5.84	33.23	655	26.13	0.76	293.33	5.68	4.74	9.68	34.77	1.04	1.34
Tongren	6.14	11.55	847.72	16.15	0.73	184.62	14.07	0.18	174.99	75.81	3.48	1.21

AN, alkali-hydrolyzable nitrogen; TN, total nitrogen; TK, total potassium; TP, total phosphorus; AK, available potassium; TOC, total organic carbon; AP, available phosphorus; AMn, available manganese; AFe, available iron; ACu, available copper; AZn, available zinc.

## References

[1] Knief C., Frances L., Vorholt J.A. (2010). Competitiveness of Diverse *Methylobacterium Strains* in the Phyllosphere of *Arabidopsis thaliana* and Identification of Representative Models, Including *M. extorquens PA_1_*. Microb. Ecol..

[2] Martirosyan V., Unc A., Miller G., Doniger T., Wachtel C., Steinberger Y. (2016). Desert Perennial Shrubs Shape the Microbial-Community Miscellany in Laimosphere and Phyllosphere Space. Microb. Ecol..

[3] Vorholt J.A. (2012). Microbial life in the phyllosphere. Nat. Rev. Microbiol..

[4] Guttman D.S., McHardy A.C., Schulze-Lefert P. (2014). Microbial genome-enabled insights into plant-microorganism interactions. Nat. Rev. Genet..

[5] Bao L.J., Cai W.Y., Zhang X.F., Liu J.H., Chen H., Wei Y.S., Jia X.X., Bai Z.H. (2020). Distinct Microbial Community of Phyllosphere Associated with Five Tropical Plants on Yongxing Island, South China Sea (vol 7, 525, 2019). Microorganisms.

[6] Romero F.M., Marina M., Pieckenstain F.L. (2016). Novel components of leaf bacterial communities of field-grown tomato plants and their potential for plant growth promotion and biocontrol of tomato diseases. Res. Microbiol..

[7] Devarajan A.K., Sabarinathan K.G., Gomathy M., Kannan R., Balachandar D. (2020). Mitigation of drought stress in rice crop with plant growth promoting abiotic stress-tolerant rice phyllosphere bacteria. J. Basic Microbiol..

[8] Carrión O., Gibson L., Elias D., Mcnamara N.P., Murrell J.C. (2020). Diversity of isoprene-degrading bacteria in phyllosphere and soil communities from a high isoprene-emitting environment: A Malaysian oil palm plantation. Microbiome.

[9] Qza C., Wla C., Yan L.B., Mk B., Qian Q.B., Wy B., Xpa B., Hqa B. (2020). Enantioselective effects of imazethapyr residues on *Arabidopsis thaliana* metabolic profile and phyllosphere microbial communities—ScienceDirect. J. Environ. Sci..

[10] Aydogan E.L., Gerald M., Christoph M., Peter K., Glaeser S.P. (2018). Long-Term Warming Shifts the Composition of Bacterial Communities in the Phyllosphere of Galium album in a Permanent Grassland Field-Experiment. Front. Microbiol..

[11] Truchado P., Gil M.I., Moreno-Candel M., Allende A. (2019). Impact of weather conditions, leaf age and irrigation water disinfection on the major epiphytic bacterial genera of baby spinach grown in an open field. Food Microbiol..

[12] Thapa S., Prasanna R. (2018). Prospecting the characteristics and significance of the phyllosphere microbiome. Ann. Microbiol..

[13] Tian L., Lin X.L., Tian J., Ji L., Chen Y.L., Tran L.S.P., Tian C.J. (2020). Research Advances of Beneficial Microbiota Associated with Crop Plants. Int. J. Mol. Sci..

[14] Wagner M.R., Roberts J.H., Balint-Kurti P., Holland J.B. (2020). Heterosis of leaf and rhizosphere microbiomes in field-grown maize. New Phytol..

[15] Jackson E.F., Echlin H.L., Jackson C.R. (2006). Changes in the phyllosphere community of the resurrection fern, Polypodium polypodioides, associated with rainfall and wetting. FEMS Microbiol. Ecol..

[16] Kadivar H., Stapleton A.E. (2003). Ultraviolet radiation alters maize phyllosphere bacterial diversity. Microb. Ecol..

[17] Yang C.H., Crowley D.E., Borneman J., Keen N.T. (2001). Microbial phyllosphere populations are more complex than previously realized. Proc. Natl. Acad. Sci. USA.

[18] Rastogi G., Sbodio A., Tech J.J., Suslow T.V., Coaker G.L., Leveau J.H.J. (2012). Leaf microbiota in an agroecosystem: Spatiotemporal variation in bacterial community composition on field-grown lettuce. ISEM J..

[19] Stone B.W.G., Jackson C.R. (2016). Biogeographic Patterns Between Bacterial Phyllosphere Communities of the Southern Magnolia (Magnolia grandiflora) in a Small Forest. Microb. Ecol..

[20] Copeland J.K., Yuan L., Layeghifard M., Wang P.W., Guttman D.S. (2015). Seasonal community succession of the phyllosphere microbiome. Mol. Plant-Microbe Interact..

[21] Samapundo S., Devliehgere F., De Meulenaer B., Debevere J. (2005). Effect of water activity and temperature on growth and the relationship between fumonisin production and the radial growth of Fusarium verticillioides and Fusarium proliferatum on corn. J. Food Prot..

[22] Laforest-Lapointe I., Messier C., Kembel S.W. (2016). Host species identity, site and time drive temperate tree phyllosphere bacterial community structure. Microbiome.

[23] Xing L., Yang J., Jia Y., Hu X., Liu Y., Xu H., Yin H., Li J., Yi Z. (2021). Effects of ecological environment and host genotype on the phyllosphere bacterial communities of cigar tobacco (*Nicotiana tabacum* L.). Ecol. Evol..

[24] Bao L.J., Gu L.K., Sun B., Cai W.Y., Zhang S.W., Zhuang G.Q., Bai Z.H., Zhuang X.L. (2020). Seasonal variation of epiphytic bacteria in the phyllosphere of *Gingko biloba*, *Pinus bungeana* and *Sabina chinensis*. FEMS Microbiol. Ecol..

[25] Joung Y.S., Ge Z.F., Buie C.R. (2017). Bioaerosol generation by raindrops on soil. Nat. Commun..

[26] Carvalho S.D., Castillo J.A. (2018). Influence of Light on Plant-Phyllosphere Interaction. Front. Plant Sci..

[27] Noble A.S., Noe S., Clearwater M.J., Lee C.K. (2020). A core phyllosphere microbiome exists across distant populations of a tree species indigenous to New Zealand. PLoS ONE.

[28] Truchado P., Gil M.I., Reboleiro P., Rodelas B., Allende A. (2017). Impact of solar radiation exposure on phyllosphere bacterial community of red-pigmented baby leaf lettuce. Food Microbiol..

[29] Wallace J., Laforest-Lapointe I., Kembel S.W. (2018). Variation in the leaf and root microbiome of sugar maple (*Acer saccharum*) at an elevational range limit. PeerJ.

[30] Rao Y., Qian Y., She X., Yang J.T., He P.H., Jiang Y.L., Wang M., Xiang W.L. (2018). Pellicle formation, microbial succession and lactic acid utilisation during the aerobic deteriorating process of Sichuan pickle. Int. J. Food Sci. Technol..

[31] Avila C.L.S., Carvalho B.F. (2020). Silage fermentation-updates focusing on the performance of micro-organisms. J. Appl. Microbiol..

[32] Borreani G., Tabacco E., Schmidt R.J., Holmes B.J., Muck R.E. (2018). Silage review: Factors affecting dry matter and quality losses in silages. J. Dairy Sci..

[33] Dong R., Tian Z. (2022). College of Animal Science, Guizhou University, Guiyang, Guizhou Province, China.

[34] Hewitt K.M., Gerba C.P., Maxwell S.L., Kelley S.T. (2012). Office space bacterial abundance and diversity in three metropolitan areas. PLoS ONE.

[35] Guo M.J., Wu F.H., Hao G.G., Qi Q., Li R., Li N., Wei L.M., Chai T.J. (2017). Bacillus subtilis Improves Immunity and Disease Resistance in Rabbits. Front. Immunol..

[36] Magoc T., Salzberg S.L. (2011). FLASH: Fast length adjustment of short reads to improve genome assemblies. Bioinformatics.

[37] Edgar R.C., Haas B.J., Clemente J.C., Quince C., Knight R. (2011). UCHIME improves sensitivity and speed of chimera detection. Bioinformatics.

[38] Edgar R.C. (2013). UPARSE: Highly accurate OTU sequences from microbial amplicon reads. Nat. Methods.

[39] Pruesse E., Quast C., Knittel K., Fuchs B.M., Ludwig W., Peplies J., Glöckner F.O. (2007). SILVA: A comprehensive online resource for quality checked and aligned ribosomal RNA sequence data compatible with ARB. Nucleic Acids Res..

[40] Krzywinski M., Schein J., Birol I., Connors J., Gascoyne R., Horsman D., Jones S.J., Marra M.A. (2009). Circos: An information aesthetic for comparative genomics. Genome Res..

[41] Grady K.L., Sorensen J.W., Stopnisek N., Guittar J., Shade A. (2019). Assembly and seasonality of core phyllosphere microbiota on perennial biofuel crops. Nat. Commun..

[42] Grobelak A., Hiller J. (2017). Bacterial siderophores promote plant growth: Screening of catechol and hydroxamate siderophores. Int. J. Phytoremediat..

[43] Cernava T., Chen X.Y., Krug L., Li H.X., Yang M.F., Berg G. (2019). The tea leaf microbiome shows specific responses to chemical pesticides and biocontrol applications. Sci. Total Environ..

[44] Thapa S., Prasanna R., Ranjan K., Velmourougane K., Ramakrishnan B. (2017). Nutrients and host attributes modulate the abundance and functional traits of phyllosphere microbiome in rice. Microbiol. Res..

[45] Xiong C., Zhu Y.G., Wang J.T., Singh B., Han L.L., Shen J.P., Li P.P., Wang G.B., Wu C.F., Ge A.H. (2021). Host selection shapes crop microbiome assembly and network complexity. New Phytol..

[46] Muller D.B., Vogel C., Bai Y., Vorholt J.A. (2016). The Plant Microbiota: Systems-Level Insights and Perspectives. Annu. Rev. Genet..

[47] Janakiev T., Dimkic I., Bojic S., Fira D., Stankovic S., Beric T. (2020). Bacterial communities of plum phyllosphere and characterization of indigenous antagonistic *Bacillus thuringiensis* R3/3 isolate. J. Appl. Microbiol..

[48] Toju H., Okayasu K., Notaguchi M. (2019). Leaf-associated microbiomes of grafted tomato plants. Sci. Rep..

[49] McGarvey J.A., Franco R.B., Palumbo J.D., Hnasko R., Stanker L., Mitloehner F.M. (2013). Bacterial population dynamics during the ensiling of *Medicago sativa* (alfalfa) and subsequent exposure to air. J. Appl. Microbiol..

[50] Zheng M.L., Niu D.Z., Jiang D., Zuo S.S., Xu C.C. (2017). Dynamics of microbial community during ensiling direct-cut alfalfa with and without LAB inoculant and sugar. J. Appl. Microbiol..

[51] Andrews J.H., Harris R.F. (2000). The Ecology and Biogeography of Microorganisms on Plant Surfaces. Annu. Rev. Phytopathol..

[52] Krishnamoorthy R., Kwon S.W., Kumutha K., Senthilkumar M., Ahmed S., Sa T., Anandham R. (2018). Diversity of culturable methylotrophic bacteria in diffrent genotypes of groundnut and their potential for plant growth promotion. J. Biotechnol..

[53] Meena K.K., Kumar M., Kalyuzhnaya M.G., Yandigeri M.S., Singh D.P., Saxena A.K., Arora D.K. (2012). Epiphytic pink-pigmented methylotrophic bacteria enhance germination and seedling growth of wheat (*Triticum aestivum*) by producing phytohormone. Antonie van Leeuwenhoek.

[54] Wipat A., Harwood C.R. (1999). The Bacillus subtilis genome sequence: The molecular blueprint of a soil bacterium. FEMS Microbiol. Ecol..

[55] Sartori M., Nesci A., Formento Á., Etcheverry M. (2015). Selection of potential biological control of *Exserohilum turcicum* with epiphytic microorganisms from maize. Rev. Argent. Microbiol..

[56] Innerebner G., Knief C., Vorholt J.A. (2011). Protection of *Arabidopsis thaliana* against Leaf-Pathogenic *Pseudomonas syringae* by *Sphingomonas* Strains in a Controlled Model System. Appl. Environ. Microbiol..

[57] Dunière L., Sindou J., Chaucheyras-Durand F., Chevallier I., Thévenot-Sergentet D. (2013). Silage processing and strategies to prevent persistence of undesirable microorganisms. Anim. Feed. Sci. Technol..

[58] Lee D.H., Kim J.-B., Kim M., Roh E., Jung K., Choi M., Oh C., Choi J., Yun J., Heu S. (2013). Microbiota on spoiled vegetables and their characterization. J. Food Prot..

[59] Ramirez-Vega H., Arteaga-Garibay R.I., Maya-Lucas O., Gomez-Rodriguez V.M., Chavez-Diaz I.F., Ruvalcaba-Gomez J.M., Heredia-Nava D., Loperena-Martinez R., Zelaya-Molina L.X. (2020). The Bacterial Community Associated with the Amarillo Zamorano Maize (*Zea mays*) Landrace Silage Process. Microorganisms.

[60] Grube M., Schmid F., Berg G. (2011). Black fungi and associated bacterial communities in the phyllosphere of grapevine. Fungal Biol..

[61] Gu L.K., Bai Z.H., Jin B., Hu Q., Wang H.L., Zhuang G.Q., Zhang H.X. (2010). Assessing the impact of fungicide enostroburin application on bacterial community in wheat phyllosphere. J. Environ. Sci..

[62] Legein M., Smets W., Vandenheuvel D., Eilers T., Muyshondt B., Prinsen E., Samson R., Lebeer S. (2020). Modes of Action of Microbial Biocontrol in the Phyllosphere. Front. Microbiol..

[63] Darlison J., Mogren L., Rosberg A.K., Gruden M., Minet A., Line C., Mieli M., Bengtsson T., Hakansson A., Uhlig E. (2019). Leaf mineral content govern microbial community structure in the phyllosphere of spinach (*Spinacia oleracea*) and rocket (*Diplotaxis tenuifolia*). Sci. Total Environ..

[64] Redford A.J., Fierer N. (2009). Bacterial Succession on the Leaf Surface: A Novel System for Studying Successional Dynamics. Microb. Ecol..

[65] Hunter P.J., Hand P., Pink D., Whipps J.M., Bending G.D. (2010). Both Leaf Properties and Microbe-Microbe Interactions Influence Within-Species Variation in Bacterial Population Diversity and Structure in the Lettuce (*Lactuca* Species) Phyllosphere. Appl. Environ. Microbiol..

[66] Kim M., Singh D., Lai-Hoe A., Rahim R.A., Ainuddin A.N., Chun J., Adams J.M. (2012). Distinctive Phyllosphere Bacterial Communities in Tropical Trees. Microb. Ecol..

[67] Chen X.Y.L., Wicaksono W.A., Berg G., Cernava T. (2021). Bacterial communities in the plant phyllosphere harbour distinct responders to a broad-spectrum pesticide. Sci. Total Environ..

[68] Redford A.J., Bowers R.M., Knight R., Linhart Y., Fierer N. (2010). The ecology of the phyllosphere: Geographic and phylogenetic variability in the distribution of bacteria on tree leaves. Environ. Microbiol..

[69] Leff J.W., Del Tredici P., Friedman W.E., Fierer N. (2015). Spatial structuring of bacterial communities within individual *Ginkgo biloba* trees. Environ. Microbiol..

[70] Guan H., Yan Y., Li X., Li X., Shuai Y., Feng G., Ran Q., Cai Y., Li Y., Zhang X. (2018). Microbial communities and natural fermentation of corn silages prepared with farm bunker-silo in Southwest China. Bioresour. Technol..

[71] Vacher C., Hampe A., Porté A.J., Sauer U., Compant S., Morris C.E. (2016). The Phyllosphere: Microbial Jungle at the Plant–Climate Interface. Annu. Rev. Ecol. Evol. Syst..

[72] Rico L., Ogaya R., Terradas J., Peñuelas J., Papen H. (2014). Community structures of N_2_-fixing bacteria associated with the phyllosphere of a Holm oak forest and their response to drought. Plant Biol..

[73] Xu J., Kloepper J.W., Huang P., McInroy J.A., Hu C.H. (2018). Isolation and characterization of N_2_-fixing bacteria from giant reed and switchgrass for plant growth promotion and nutrient uptake. J. Basic Microbiol..

[74] Paul K., Saha C., Nag M., Mandal D., Naiya H., Sen D., Mitra S., Kumar M., Bose D., Mukherjee G. (2019). A Tripartite Interaction among the Basidiomycete *Rhodotorula mucilaginosa*, N_2_-Fixing Endobacteria, and Rice Improves Plant Nitrogen Nutrition. Plant Cell.

[75] Kaymak H.C., Aksoy A., Kotan R. (2020). Inoculation With N_2_-Fixing Plant Growth Promoting Rhizobacteria to Reduce Nitrogen Fertilizer Requirement of Lettuce. Acta Sci. Pol. Hortorum Cultus.

[76] Vissers M., Giffel M., Driehuis F., Jong P.D., Lankveld J. (2007). Minimizing the level of Bacillus cereus spores in farm tank milk. J. Dairy Sci..

[77] Dayton P.K. (1995). Biological diversity: The coexistence of species on changing landscapes. J. Exp. Mar. Biol. Ecol..

